# Emerging players in the initiation of eukaryotic DNA replication

**DOI:** 10.1186/1747-1028-7-22

**Published:** 2012-10-17

**Authors:** Zhen Shen, Supriya G Prasanth

**Affiliations:** 1 Department of Cell and Developmental Biology, University of Illinois at Urbana-Champaign, 601 S. Goodwin Avenue, Urbana, IL 61801, USA

**Keywords:** ORC, ORCA/LRWD1, Cdt1, Geminin, MCM, Pre-RC, Pre-IC, RPC, Non-coding RNA, DNA replication

## Abstract

Faithful duplication of the genome in eukaryotes requires ordered assembly of a multi-protein complex called the pre-replicative complex (pre-RC) prior to S phase; transition to the pre-initiation complex (pre-IC) at the beginning of DNA replication; coordinated progression of the replisome during S phase; and well-controlled regulation of replication licensing to prevent re-replication. These events are achieved by the formation of distinct protein complexes that form in a cell cycle-dependent manner. Several components of the pre-RC and pre-IC are highly conserved across all examined eukaryotic species. Many of these proteins, in addition to their bona fide roles in DNA replication are also required for other cell cycle events including heterochromatin organization, chromosome segregation and centrosome biology. As the complexity of the genome increases dramatically from yeast to human, additional proteins have been identified in higher eukaryotes that dictate replication initiation, progression and licensing. In this review, we discuss the newly discovered components and their roles in cell cycle progression.

## Introduction

The proper inheritance of genomic information in eukaryotes requires both well-coordinated DNA replication in S phase and separation of duplicated chromosomes into daughter cells in mitosis [1]. Prior to S phase, pre-replication complex (pre-RC), a multi-protein complex which dictates when and where the DNA replication will initiate, is assembled [2-6]. Studies in *Saccharomyces cerevisiae* revealed conserved replication initiation sites (origins) that comprise a highly conserved autonomously replicating sequence (ARS) [7]. Identification of proteins bound to this sequence led to the discovery of a six-subunit complex that serves as the initiator to select replication initiation sites, and was therefore named the origin recognition complex (ORC) [8]. The assembly of pre-RC starts with ORC recognizing the replication elements and recruiting two factors, Cdc6 and Cdt1. These proteins function together to load the minichromosome maintenance proteins (MCM) onto chromatin [2-6]. This process takes place as early as the end of mitosis of the previous cell cycle [9]. In yeast, at the onset of S phase, Dbf4-dependent kinase (DDK) phosphorylation of MCMs and cyclin-dependent kinases (CDKs) phosphorylation of Sld2 and Sld3 lead to the assembly of Dpb11, GINS complex, MCM10, Cdc45, and DNA polymerase to initiation sites to form the pre-initiation complex (pre-IC), which in turn activates the MCM helicase [1-4,10,11]. In higher eukaryotes, a similar cascade has been identified, with RecQ4 and TopBP1 being orthologs for Sld2 and Dpb11 respectively [1,11]. In order to maintain the genome content, replication must occur “once and only once” during each cell cycle and re-replication must be strictly prevented. This “replication licensing” mission is carried out by multiple mechanisms at the levels of the regulation of mRNA transcription, protein localization and protein stability, the presence of pre-RC inhibitors, and the alteration of local chromatin architecture [3,4,6,12-14].

Since the initial identification of ORC in *Saccharomyces cerevisiae* in 1992 [8], tremendous progress has been made in the past two decades in dissecting how the assembly of pre-RC and pre-IC regulates the initiation event of DNA replication. The ordered assembly has been found to be highly conserved in all the examined model organisms, including budding and fission yeast, *Drosophila*, *Xenopus*, and mammalian cells. However, as the complexity of the organisms increases dramatically, significant differences between organisms become apparent. For example, the ortholog for Sld3 in higher eukaryotes is missing. It is also clear that additional mechanisms control the initiation and completion of replication in higher eukaryotes. For instance, Geminin, the pre-RC inhibitor, can only be found in metazoans [15,16]. In recent years, many new factors including several proteins and RNAs have been unveiled to play important roles in pre-RC/pre-IC assembly and licensing. In this review, we survey these emerging players, categorizing them as the pre-RC/pre-IC accessory proteins to denote the proteins with which they associate. It is noteworthy that many of them are only present in higher eukaryotes.

## ORC accessory factors

The primary role of ORC is sequence identification and origin binding. During pre-RC assembly, ORC binding to origins serves as the landing pad for the sequential loading of Cdc6, Cdt1, and MCM2-7 to these sites [4]. However, even though some specific DNA binding sites have been revealed in different eukaryotic organisms, no consensus has been identified other than the ACS (ARS consensus sequence) in the budding yeast. In addition, though ORC is conserved in eukaryotes, the mechanism that recruits ORC to chromatin remains to be clearly elucidated in metazoans [4]. The discovery of ORC-associating factors may help us fill the missing blocks.

### ORCA/LRWD1

ORCA/LRWD1 (ORC associated/leucine-rich repeats and WD repeat domain containing 1) was identified from the mass spectrometric analysis of ORC-interacting proteins [17]. It co-localizes with ORC at heterochromatic sites and shows similar cell cycle dynamics to that of ORC in human cells. Tethering ORCA to an artificially generated chromatin region efficiently recruits ORC to that chromatin locus [17]. Further, depletion of ORCA in human primary cells and embryonic stem cells results in the loss of ORC association to chromatin, the reduction of MCM binding to chromatin, and the subsequent accumulation of cells in G1 phase [17]. These data suggest that ORCA is required for the stable association of ORC to chromatin.

ORCA protein levels fluctuate throughout the cell cycle, peaking in G1 phase [18]. In addition to ORC, ORCA also associates with other cell cycle regulated proteins: Cdt1 and Geminin, and these interactions are cell cycle-dependent: ORCA associates with ORC core complex throughout the cell cycle, with Cdt1 in mitosis and G1, and with Geminin in post-G1 phases [18]. Single molecule analyses demonstrate that one molecule of ORCA binds to one ORC, one Cdt1 and/or two Geminin [18]. Overexpression of Geminin in human cells results in the loss of interaction between ORCA and Cdt1, suggesting that increased levels of Geminin in post-G1 cells titrate Cdt1 away from ORCA [18]. Taken together, these data suggest that ORCA modulates the stoichiometric assembly of the pre-RC components, and may serve as another licensing factor, in addition to its role in facilitating pre-RC assembly on chromatin.

Structural and functional analysis reveals that the five WD repeats of ORCA are essential for its interaction with ORC, Cdt1, Geminin, and its chromatin association [17-20]. The WD domain forms a circular β-propeller structure, with each repeat unit as a blade in the form of a β sheet [21-24]. Therefore, the intact WD domain is important for these functional associations, as deletion of any repeat abolishes the interaction [18,20]. The N-terminus of Orc2 interacts directly with the WD domain of ORCA [18]. Upon Orc2 depletion, ORCA also undergoes rapid destruction, which can be rescued by the addition of proteasome inhibitor MG132, indicating that Orc2 protects ORCA from ubiquitin-mediated degradation. This is supported by the fact that K48 polyubiquitin chain can be formed on the WD domain of ORCA, and Orc2 only interacts with the non-ubiquitinated form of ORCA [25].

Notably, ORCA also associates with heterochromatic regions and has been shown to bind to repressive histone marks [19,20,26]. ORCA associates with H3K9me3, H4K20me3, and H3K27me3 peptides and is recruited to pericentric heterochromatin through its association with H3K9me3 [20]. Moreover, ChIP-seq using the BAC ORCA-GFP cell line displays an enriched signal on satellite repeats [26], and depletion of ORCA in MEF cells results in the up-regulation of major satellite repeat transcripts, suggesting its requirement for heterochromatin silencing [20]. Therefore, ORCA may function as a key molecule that links pre-RC assembly to higher order chromatin structure [27].

Interestingly, ORCA and ORC localize to centromeres in human mammary epithelial cells (MCF7) and other telomerase positive cell lines throughout the cell cycle [17,28]. A proteomic screen of mitotic chromosome-associated proteins also identified ORCA (alias CENP-33) as a novel centromere protein [29]. These observations indicate the possible involvement of ORCA in DNA recombination, since centromeres are highly recombinogenic regions [30]. ORCA was found to localize to telomeres in interphase U2OS (Osteosarcoma) cells [17], which utilize DNA recombination mediated ALT mechanism (Alternative Lengthening of Telomeres) to maintain their telomeres [31]. ORCA was also identified in a large-scale proteomic analysis of ATM/ATR substrates as one of the proteins that is phosphorylated in response to DNA damage, corroborating ORCA’s role in DNA recombination/repair [32]. Moreover, the mouse ortholog of ORCA is highly expressed in the testis, an organ with high recombinogenic activity, and has been reported to be involved in spermatogenesis [33]. Further investigation on the functional significance of ORCA binding to centromeres and telomeres will be critical to address if ORCA has a direct role in heterochromatin replication or heterochromatin organization and if ORCA plays any role in DNA repair/recombination.

### HBO1

HBO1 (human acetylase binding to Orc1) was originally identified in a yeast two-hybrid screen from a HeLa cell cDNA library using Orc1 as the bait. The histone acetyltransferase activities were found in the HBO1-containing complex, indicating that an ORC-mediated chromatin acetylation influenced DNA replication [34]. In human cells, HBO1 knockdown leads to the MCM chromatin loading defect, with no effect on chromatin loading of ORC and Cdc6, suggesting HBO1’s involvement at a step after ORC and Cdc6 binding to chromatin but upstream of MCM loading. In *Xenopus* egg extracts, immunodepletion of HBO1 also impairs chromatin binding of MCM and inhibits DNA replication, but this can be restored upon the addition of recombinant Cdt1 [35].

HBO1 associates with origins in G1 phase, directly interacts with Cdt1, and enhances Cdt1-dependent re-replication [36]. It has been suggested that HBO1 acts as the co-activator of Cdt1 and thereby facilitates replication initiation [36]. Further, HBO1-mediated histone H4 acetylation at origins is required for MCM loading, and Geminin inhibits HBO1 acetylase activity in a Cdt1-dependent manner [37]. This is consistent with a recent report that Cdt1-HBO1 complex promotes MCM loading through acetylation-mediated enhancement of chromatin accessibility in G1 phase. The MCM loading is inhibited by Cdt1-Geminin-HDAC11 via deacetylation in S phase, providing yet another mechanism for replication licensing [38]. Interestingly, Cdt1-HBO1 interaction is well regulated: in response to stress, JNK1 phosphorylates Cdt1 on threonine 29, which results in the dissociation of HBO1 from replication origins and consequently results in the inhibition of replication initiation [39]. Taken together, HBO1 is a key molecule that organizes chromatin to facilitate pre-RC assembly and replication initiation.

### 14-3-3

14-3-3 proteins exhibit specific phospho-serine/phospho-threonine binding activities, and thus are involved in various cellular pathways, including cell growth, apoptosis, cytokinesis, and tumor suppression [40,41]. In mammalian cells, CBP (cruciform-binding protein) belongs to the 14-3-3 family. ChIP experiments reveal that CBP associates with monkey replication origins *ors8* and *ors12*, which bear inverted repeats and form the cruciform structure. This origin-association takes place in a cell cycle-dependent manner, with maximal activity at the G1/S boundary [42-44]. Addition of CBP antibodies impairs CBP-cruciform DNA complex formation and inhibits DNA replication *in vitro*[42,43]. In *Saccharomyces cerevisiae*, Bmh1 and Bmh2, the 14-3-3 homologs in budding yeast, also have cruciform DNA binding activities and bind to replication origin ARS307 *in vivo*[45,46]. Recently, the role of 14-3-3 in replication initiation has been elucidated. Bmh2 interacts with Orc2 and MCM2, and binds to ARS, peaking in G1 phase [47]. Utilizing a Bmh2 temperature-sensitive (*bmh2-ts*) mutant strain, it has been demonstrated that Bmh2 is required for the loading and maintenance of MCM on chromatin during G1 phase. Bmh2 has been suggested to be an essential component for pre-RC formation, DNA replication initiation, and normal cell cycle progression [47]. 14-3-3 binding to cruciform DNA indicates that additional factors may be needed in eukaryotes for replication activation at specific DNA structures.

This site-specific association of ORC accessory factors is also evident at other DNA contexts. In *Drosophila*, the Myb (Myeloblastosis)-containing complex (Myb p85, Caf1 p55, p40, p120, and p130) binds in a site-specific manner to the chorion gene cluster, ACE3 and *ori-β*. The Myb complex interacts with ORC, and is essential for chorion gene amplification [48]. Myb may function in converting specific inactive replication origins to active ones, possibly by facilitating acetylation at origins [49,50]. In mammalian cells, high mobility group (HMG) proteins have the AT-hook motif that binds to the minor groove of the AT-rich regions of double-stranded DNA [51]. A member of the HMG proteins, HMGA1a, associates with ORC *in vitro* and *in vivo*[52]. Targeting HMGA1a to specific DNA sites recruits ORC and creates functional replication origins, and the abundance of recruited ORC correlates with the local density of HMGA1a. It is therefore proposed that high local concentration of HMGA1a may function as a potent, dominant replication origin [52]. These examples indicate that it will be critical to determine if a defined set of protein complexes associates with specific DNA elements that in turn regulates the replication initiation at site-specific replication origins.

## Cdt1-Geminin associated proteins

Cdt1 is required for loading MCM onto origins, and it accumulates within the nucleus and associates to chromatin with high expression levels only in G1. In metazoan, another cell cycle regulated protein, Geminin, accumulates during S-G2-M phases and is then mitotically degraded by APC/C (anaphase promoting complex/cyclosome). Geminin is an inhibitor of Cdt1 and does not allow the assembly of pre-RC outside of G1 phase. Therefore, the dynamic oscillating pattern ensures that pre-RC is assembled only in G1 phase and is strictly inhibited outside G1 phase, serving as one of the replication licensing mechanisms that is critical for the maintenance of genome stability [4].

### HOX

HOX proteins control cell fate determination and patterning specification processes during development. They function as transcription factors that bind to DNA via their homeodomain [53-57]. Through a yeast two-hybrid screen using a cDNA library from 8.5 days post-coitum mouse embryos, Geminin was found to associate with HOX proteins, the HOX regulatory DNA elements, as well as the HOX-repressing polycomb complex [58]. Geminin inhibits the transcriptional activator function of HOX. HOX binding to Geminin impairs the association of Cdt1 with Geminin [58]. Interestingly, HOXD13, HOXD11 and HOXA13 bind to human replication origins *in vivo*; and HOXD13 directly interacts with Cdc6 via its homeodomain. This association promotes pre-RC assembly at origins and eventually stimulates DNA replication [59]. Binding of Geminin to HOXD13 blocks its pre-RC promoting function; however, exogenous HOXD13 expression overrides the Geminin-induced G1 accumulation [59]. A recent study has resolved the solution structure of the homeodomain of HOXC9 (HOXC9-HD) in complex with Geminin homeodomain binding region (Gem-HBR). Interestingly, the C-terminal Ser184 residue of Geminin can be phosphorylated by Casein kinase II (CK2), resulting in its higher binding affinity and inhibitory effect toward HOX [60]. Taken together, these data establish the HOX interactions with pre-RC and Geminin as an additional mechanism for controlling pre-RC assembly and replication initiation.

### Idas

Discovered as a Geminin homolog, Idas is highly similar with Geminin’s central coiled-coil region. The term “Idas” was derived after the name of Gemini’s cousin (in ancient Greek mythology). In human cells, Idas localizes in the nucleus and shows decreased protein levels in anaphase [61]. Idas forms a complex with Geminin, but not Cdt1, and this direct binding prevents Geminin from binding to Cdt1 and translocates Geminin from the cytoplasm to the nucleus [61]. Depletion of Idas leads to the accumulation of cells in S phase and inefficient progression to mitosis and G1, whereas the over-expression of Idas leads to accumulation of multi-nucleated cells [61]. In contrast, over-expression of Geminin shows S phase accumulation [62], while depletion of Geminin leads to multipolar spindle defects [63]. It remains to be determined if Idas has a role in replication initiation. A recent study has also indicated specific high expression of Idas in the cortical hem and choroid plexus of the developing mouse telencephalon, indicating its involvement in developmental control as a putative modulator of proliferation-differentiation determination during development [61].

Geminin has dual functions: replication licensing inhibition and developmental control [64-66]. Both HOX proteins and Idas affect normal cell cycle proliferation and are also involved in the developmental regulation of differentiation. Also notably, they both compete with Cdt1 for Geminin binding, suggesting that these proteins play crucial roles in pre-RC regulation or by balancing Geminin’s dual functions along with Cdt1.

## MCM-related proteins

MCM (minichromosome maintenance) proteins were first isolated in yeast mutants that were defective in the maintenance of circular minichromosomes in *S. cerevisiae*[67]. MCM2-7 is generally believed to serve as the DNA helicase during replication. It forms a hexameric ring structure and all of the six subunits belong to the AAA+ family (ATPases associated with a variety of cellular activities) [68]. Recently, a number of MCM related proteins were identified based on either the sequence homology or the association with MCM2-7.

### MCM8

MCM8 was categorized as a new member of the MCM family because it contained a conserved MCM helicase ATP binding domain--similar to what is observed in the MCM2-7 proteins [69,70]. In human cells, MCM8 binding to chromatin is cell cycle-regulated [71]. It interacts with Orc2 and Cdc6 [71], and co-localizes with Cdc6 at the c-*myc* initiation site [72]. Depletion of MCM8 affects the normal G1/S transition and leads to loading defects of Cdc6 and MCM onto chromatin [71]. In *Xenopus*, MCM8 has been shown to function as a DNA helicase during replication elongation, but not for replication initiation [73]. MCM8 is not required for MCM2-7 chromatin loading, but instead binds to chromatin at the onset of DNA replication, after replication licensing [73]. MCM8 co-localizes with replication foci; with its DNA helicase and DNA-dependent ATPase activities, MCM8 regulates the chromatin assembly of DNA polymerase α and RPA34 [73]. In *Drosophila* S2 cells, MCM8 depletion diminishes PCNA binding by 30–50%, also indicating the involvement of MCM8 in DNA synthesis [74].

Different cell assay systems and species might explain the differences regarding the roles of MCM8 in replication initiation and/or elongation. In *Xenopus*, Cdc6 displays reduced chromatin association after replication licensing, and is reloaded onto chromatin at the onset of S phase [75]. In HeLa cells, MCM8 and Cdc6 co-localize at the c-*myc* replication initiation zone during G1 and this association continues even after completion of DNA replication [72]. These data indicate that the loss of Cdc6 association to chromatin in MCM8-depleted cells could either suggest a role of MCM8 in loading Cdc6 onto chromatin to facilitate pre-RC assembly, and/or an independent role in post-G1 phase cells. Moreover, during a small time window at the G1/S boundary, the chromatin bound levels of MCM8 drop significantly, thus manifesting two discontinuous functions of MCM8 and possibly the dual role in both DNA replication initiation and elongation [72].

### MCM9

MCM9 was identified as another MCM family member by bioinformatic approaches [76,77]. In *Xenopus*, the binding of MCM9 onto chromatin is dependent on ORC. Further, MCM9 facilitates the loading of MCM2-7 onto chromatin [78]. In the absence of MCM9, pre-RC assembly is hampered and DNA replication halts. Mechanistic analysis shows that MCM9 forms a complex with Cdt1, limiting the amount of Geminin that can associate with Cdt1 on chromatin during replication licensing. This enables the active Cdt1 to load MCM2-7 complex onto chromatin by modulating the ratio of Cdt1 to Geminin [78,79]. However, MCM9 is not required for pre-RC formation or DNA replication in mice, as no change in the chromatin-bound MCM2/4/7 or Cdt1 was observed upon MCM9 disruption [80]. It is possible that these organisms have evolved species-specific and tissue-specific requirements.

Recently, using MCM8 and MCM9 knockout chicken DT40 cell lines as well as MCM8−/− and MCM9−/− deficient mice, two groups demonstrated that MCM8 and MCM9 form a complex, distinct from the MCM2-7 complex. Further MCM8 and MCM9 complex co-regulate their stability. Importantly, they both play essential roles in homologous recombination-mediated double strand break repair during replication fork maintenance [81,82].

### MCM10

MCM10 was identified in genetic screens for mini-chromosome maintenance defects and DNA replication defects [67,83-85]. It does not exhibit sequence homology to MCM2-7, but is conserved in most eukaryotes [86], and it also has the ability to bind both single and double stranded DNA [87-92]. In *Saccharomyces cerevisiae* and *Xenopus laevis*, MCM10 can self-assemble into a homo-complex, which requires its zinc finger motif [89,93]; and in human cells, MCM10 forms a hexameric ring structure [88]. MCM10 interacts with MCM2-7, which is essential for DNA replication initiation in all eukaryotes examined, including *Saccharomyces cerevisiae*[85,94,95], *Schizosaccharomyces pombe*[96-98], *Xenopus*[99], *Drosophila*[100], and human cells [99,101]. Chromatin loaded MCM2-7 (the inactive form) enables the efficient recruitment of MCM10 [102]. MCM10 also interacts with DNA polymerase α [87,89-91,99,103-106] and recruits DNA polymerase α to replication origins/forks and stabilizes its catalytic subunit [103-105], functioning together with Ctf4/And-1 (see below the Ctf4/And-1 section) [99,107]. Taken together, MCM10 may serve as the coordinator for MCM2-7 helicase and DNA polymerase, facilitating their roles during replication initiation and elongation.

Even though the role of MCM10 in DNA replication has been appreciated for a while, significant new investigations into its interaction with the Cdc45-MCM2-7-GINS (CMG) complex and the functional significance of this has prompted us to include MCM10 in the review. MCM10 binds to origins in a TopBP1 dependent manner [108] and associates with replication initiation sites before Cdc45, promoting Cdc45 chromatin association [109-111], as well as after Cdc45 along with DNA polymerase α [103,112]. This pre- and post- Cdc45 recruitment of MCM10 is intriguing, indicating its complicated but yet uncovered function with CMG.

Recently, several groups have reported that the origin association of MCM10 is independent of CMG assembly, but is essential for DNA unwinding [102,113,114]. In *Saccharomyces cerevisiae*, the stable CMG complex remains at origins upon the auxin-induced MCM10 degradation (*mcm10-1-aid*) [113]. Further, Cdc45-MCM2-7-GINS interactions are not impaired upon temperature-induced MCM10 degradation (*mcm10-1td*) [102], indicating CMG origin assembly is independent of MCM10. However, RPA loading is defective in the absence of MCM10 in both studies, suggesting that MCM10 facilitates an origin DNA unwinding reaction [102,113]. Parallel observations were also made in *Schizosaccharomyces pombe* using combined promoter shut-off and auxin-induced degradation system (*off-aid*) [114]. Moreover, point mutations within the zinc finger motif in fission yeast MCM10 (MCM10^ZA^) revealed that this motif plays an important role in unwinding the origin, though it is not essential for MCM10 origin association [114].

### MCM-BP

MCM-BP (MCM-binding protein) was first identified from mass spectrometric analyses of TAP (tandem affinity purification) tagged MCM6 and MCM7 immunoprecipitations [115]. Interestingly, MCM-BP interacts with MCM3-7 but not MCM2, indicating that MCM-BP and MCM2 form different complexes with MCM3-7 [115-118]. Further, MCM-BP can interact with the core complex MCM4/6/7 *in vitro*, but does not inhibit the helicase activity [115].

In *Arabidopsis thaliana*, the MCM-BP ortholog, ETG1 (E2F target gene 1) is required for DNA replication [119]. ETG1-deficient plants display the activation of DNA replication stress checkpoint, which is crucial to their survival [119]. In *Schizosaccharomyces pombe*, overexpression of Mcb1 (MCM-BP homolog) disrupts MCM2 association with MCM3-7 complex and causes MCMs to dissociate from the chromatin, leading to DNA replication inhibition, DNA damage and checkpoint activation [117,118]. In *Xenopus* egg extracts, MCM-BP accumulates in the nucleus in late S phase. Immunodepletion of MCM-BP inhibits replication-coupled MCM2-7 dissociation from chromatin, whereas addition of excess recombinant MCM-BP disassembles the MCM2-7 complex [116]. Similar observations on these species were also obtained in human cells. MCM-BP binds to chromatin in a cell cycle-dependent manner: associating with chromatin in M-G1-S and dissociating from chromatin in late G2-early M. The MCM-BP dynamic patterning occurs slightly later than that of MCM2-7 [115]. MCM-BP siRNA knockdown results in the reduced dissociation of MCM from chromatin, consistent with its role as the MCM2-7 unloader from chromatin [116]. ShRNA prolonged knockdown results in G2 checkpoint activation, and raised whole cell levels as well as soluble levels of MCM proteins [120]. Interestingly, yeast two-hybrid assay and immunoprecipitation analysis reveal that MCM-BP also interacts with Dbf4. However, MCM-BP is not a DDK substrate; rather, it inhibits phosphorylation of MCM by DDK instead, adding another layer of complexity in replication regulation [121].

Moreover, MCM-BP is also involved in other cellular events. In *Arabidopsis thaliana*, ETG1 is required for the establishment of sister chromatid cohesion [122]; in fission yeast, MCM-BP is essential for meiosis [118]; and in human cells, MCM-BP shRNA knockdown leads to abnormal nuclear morphology and centrosomal amplification [120]. Further investigations are needed to distinguish whether these observations indicate its separate functions in DNA replication and mitosis or an indirect effect due to aberrant DNA replication.

## Factors influencing pre-IC

The establishment of pre-IC sets the stage for the initiation of DNA replication during S phase of cell cycle [4]. In addition to Cdc45, MCM10, GINS and other related proteins, several novel components have been identified that influence the pre-IC formation.

### DUE-B

DUE-B (DUE-binding protein) was identified in a yeast one-hybrid screen to identify proteins that bind to c-*myc* DUE (DNA-unwinding element)/ACS region [123]. Structural analysis reveals that DUE-B interacts with DNA via its C-terminus and forms a homodimer via the N-terminus, resembling the structure of tRNA-editing enzymes [124]. The N-terminus of DUE-B exhibits both D-aminoacyl-tRNA deacylase and ATPase activity [124]. In HeLa cells, RNAi knockdown of DUE-B results in the delayed S phase entry and increased cell death [123]. In *Xenopus* egg extracts, immunodepletion of DUE-B inhibits replication, which can be restored by the addition of recombinant DUE-B (expressed in HeLa cells) [123]. Further, DUE-B forms a complex with TopBP1 and Cdc45. Knockdown of DUE-B in HeLa cells abolishes the chromatin binding of Cdc45, and immunodepletion of DUE-B in *Xenopus* egg extracts abolishes the loading of both Cdc45 and TopBP1 to chromatin [125]. DUE-B can be phosphorylated, and this affects Cdc45 (not TopBP1) association with phosphorylated DUE-B at high salt concentration [125]. It is therefore hypothesized that TopBP1-DUE-B-Cdc45 complex associates with the pre-RC. CDK phosphorylation then triggers Cdc45-mediated activation of pre-IC [125].

### GEMC1

GEMC1 (Geminin coiled-coil containing protein 1) was identified during the search for open reading frames (ORFs) containing degenerate signature motifs present in known replication factors [126]. In *Xenopus* egg extracts, GEMC1 interacts with Cdc45 and TopBP1, and its chromatin binding is independent of the MCM2‑7 complex, suggesting its involvement subsequent to the formation of pre-RC. GEMC1 depletion prevents Cdc45’s chromatin loading, whereas TopBP1 depletion prevents GEMC1’s chromatin loading, indicating the sequential assembly where TopBP1-dependent loading of GEMC1 to chromatin promotes the subsequent loading of Cdc45 to chromatin [126]. Moreover, GEMC1 interacts with and is phosphorylated by Cyclin E/Cdk2. Constitutively phosphorylated GEMC1 stimulated efficient DNA replication and enhanced the loading of Cdc45 to chromatin [126]. These data together suggest that TopBP1- and Cdk2-dependent binding of GEMC1 to chromatin promotes the loading of Cdc45 onto chromatin to facilitate DNA replication [126,127].

### Treslin/Ticrr

Treslin was recently identified as a TopBP1 interacting protein in *Xenopus* egg extracts. Phosphorylation of Treslin by Cdk2 is required for Treslin-TopBP1 complex formation [128]. Immunodepletion of Treslin does not affect TopBP1 binding to chromatin and vice versa. Treslin and TopBP1 co-operate in the Cdk2-mediated loading of Cdc45 to chromatin [128]. Treslin phosphorylation mediated by Cdk2 has been suggested to promote the formation of a TopBP1-Treslin complex, which in turn would enable Cdc45 loading and the subsequent action of replicative helicase activity [128]. In human cells, a similar Cdk2-mediated Treslin-TopBP1 association is demonstrated [129], and Treslin knockdown is found to affect S phase progression due to accumulation of DNA damage [128]. Treslin was concurrently identified from a screen in zebrafish for G2/M checkpoint regulators, and hence named as Ticrr (TopBP1-interacting, checkpoint, and replication regulator) [130]. Ticrr, also a chromatin associated protein that binds TopBP1, has been shown to be required for the proper assembly of the pre-IC, but not the pre-RC [130].

DUE-B, GEMC1, and Treslin/Ticrr all associate with TopBP1 as well as Cdc45, and phosphorylation of these three proteins are all required for the proper activation of Cdc45 on chromatin. The formation of pre-IC requires CMG and other replication factors. In yeast, these factors belong to, but are not limited to, the Sld2-Dpb11-Sld3 complex. In higher eukaryotes, RecQ4 is generally considered the ortholog for Sld2 [131,132], and the TopBP1 for Dpb11 [133-135]. However, the counterpart for Sld3 is missing. Given the fact that CDK-mediated regulations of DUE-B, GEMC1, and Treslin/Ticrr interactions with TopBP1 and Cdc45 are similar to that of Sld3–Dbp11, it is tantalizing to propose that one or more of them may serve as the functional orthologs. Specifically, Treslin/Ticrr interacts with BRCT repeats of TopBP1, resembling that of Sld3 to Dpb11 [128-130], and conserved CDK sites between Sld3 and Treslin/Ticrr were identified; it is therefore likely that Treslin/Ticrr is functionally equivalent to Sld3 [136-139]. Nevertheless, a recent study found that neither Treslin/Ticrr, nor the above-mentioned DUE-B or GEMC1, could complement the loss of Sld3 (fail to rescue the growth defect of *sld3* mutants) in *Schizosaccharomyces pombe*, indicating much more complexity within the higher eukaryotes [140].

Interestingly, existing components in the pre-IC also exhibit functions coupling both replication initiation and elongation. In human cells, RecQ4 is loaded onto chromatin in late G1 phase after pre-RC formation, following which RecQ1 and additional RecQ4 are recruited to origins at the onset of DNA replication [141]. During DNA synthesis, RNAi knockdown of RecQ1 impairs replication fork progression [141], whereas RecQ4 displays DNA unwinding activity and functions along with CMG and MCM10 [142-144]. Our quest towards identifying novel components that regulate replication initiation and elongation continues.

## Replisome Progression Complex (RPC) coupled factors

### FACT

FACT (facilitates chromatin transcription) was first purified from HeLa cell nuclear extracts as the factor required for transcription elongation [145,146]. It interacts with nucleosomes and histone H2A/H2B dimers, and is composed of Spt16/Cdc68 and SSRP1 (structure-specific recognition protein-1) [147]. Similar findings were also reported from yeast FACT Spt16-Pob3-Nhp6 [148,149] and *Xenopus* FACT DUF (DNA unwinding factor, DUF140-DUF87) [150]. In yeast, FACT interacts with DNA polymerase and RPA [151-153], whereas in *Xenopus* egg extracts, FACT unwinds DNA and is crucial for DNA replication [150].

Interestingly, FACT interacts with MCM2-7 and promotes its DNA unwinding activity [154]. ChIP assays reveal that FACT and MCM2-7 co-localize at replication origins in human cells, and disruption of FACT-MCM interaction by introducing mutant MCM4_120-250_ results in reduced levels of FACT at origins and delayed replication initiation [154]. These data clearly demonstrate that the FACT-MCM interaction is essential for the initiation stage of DNA replication. Further, FACT-MCM interaction exhibits cell cycle regulated dynamics, with interaction levels peaking at the onset of DNA replication and during S phase [155], and the study from SSRP1 conditional knockout chicken DT40 cells indicates that FACT maintains the normal elongation rate/fork progression of DNA synthesis [156]. This suggests that FACT-MCM interaction functions not only in the initiation phase but also the elongation stage of DNA replication. Similarly in *Xenopus* egg extracts FACT displays two phases of chromatin binding: one before origin licensing and the other binding during replication [157].

### Ctf4/And-1

Ctf4 gene was first described from two genetic screens for chromosome transmission fidelity (CTF) and chromosome loss (CHL) in *Saccharomyces cerevisiae*[158], and was found essential for sister chromatid cohesion [159]. Later, it was found that Ctf4 depletion in the *orc2-1* and *orc5-1* mutants background leads to synthetic lethality, indicating its involvement in DNA replication [160]. Recent studies in several model organisms have provided more insights into the mechanism of how Ctf4 facilitates DNA replication.

In *Saccharomyces cerevisiae*, Ctf4 binds to chromatin predominantly in S phase, and this chromatin binding requires its interaction with MCM10. Deletion of Ctf4 results in the destabilization of MCM10 and DNA polymerase α, and defects in S phase [107]. Further, Ctf4 interacts directly with GINS and DNA polymerase α [161], and the interaction between CMG complex and DNA polymerase α is abolished in a *ctf4Δ* strain [162]. In *Drosophila*, Ctf4 interacts with Psf1, Psf2, MCM2, and DNA polymerase α in yeast two-hybrid analyses. *In vivo* RNAi knockdown of Ctf4 demonstrates its requirement for normal S phase progression during homeostasis as well as replication stress induced by replication fork pausing [163]. In *Xenopus*, immunodepletion of Ctf4 from egg extracts abolishes DNA replication. Time course analysis indicates that MCM10 loading onto chromatin precedes that of Ctf4 and DNA polymerase α, and MCM10-Ctf4 interaction is required for the loading of Ctf4 and DNA polymerase α [99]. In mammalian cells, Ctf4 also interacts with MCM10 and DNA polymerase, and is required for the stabilization of DNA polymerase and replication [99,164]. RNAi knockdown of Ctf4 in HeLa cells results in the failure of the CMG complex assembly on chromatin [144], and a slower DNA replication rate [164]. Therefore, Ctf4 is an essential factor in DNA replication and facilitates replisome progression [161].

The initial identification of FACT and Ctf4 indicated their functions in regulating chromatin contexts, not DNA synthesis; however, recent data have clearly demonstrated the close crosstalk between DNA replication and its chromatin context in S phase. It is noteworthy that DNA replication components act in concert with genome stability surveillance factors. For instance, the checkpoint mediator proteins Mrc1-Tof1-Csm3, as RPC components, are involved in replisome progression. In *Saccharomyces cerevisiae*, Mrc1 is required for normal replication fork movements, whereas Tof1 is necessary for replication forks to pause at protein-DNA barriers [165]. In *Schizosaccharomyces pombe*, Mrc1 regulates early-firing origins, independent of its checkpoint function [166]. Another example comes from *Xenopus* Dna2 [167,168], which co-localizes with RPA during replication and interacts with Ctf4/And-1 and MCM10. On the other hand, Dna2 also associates with DSB repair and checkpoint proteins Nbs1 and ATM [167]. These concerted interactions at the replication fork ensure genome stability during DNA synthesis.

## Non-coding RNAs

### Y RNAs

Y RNAs are conserved small stem-loop RNAs, first identified as the cytoplasmic RNA components of Ro ribonucleoprotein particles (Ro RNPs) in higher eukaryotes. In human cells, there are 4 types of Y RNAs: Y1, Y3, Y4, and Y5 [169-174]. In human cell free system (late G1 phase nuclei), both reconstitution and individual degradation of Y RNAs demonstrate that they are required for chromosomal DNA replication, and can substitute for each other [175]. However, Y1 RNA binding to Ro60 is not necessary for DNA replication [175], neither is the Y RNAs containing ribonucleoprotein complex [176]. During the investigation on how Y RNAs are involved in DNA replication, DNA combing/fiber fluorescence and nascent-strand analysis were utilized to study the effect of Y RNA degradation (Y3) and the supplementation of non-targeted Y RNA (Y1), which demonstrated that Y RNAs are required for the initiation rather than the elongation of DNA replication [177]. Interestingly, only vertebrate Y RNAs are able to reconstitute chromosomal DNA replication *in vitro*, which led to the discovery of a conserved motif in the double-stranded stem of vertebrate Y RNAs that is sufficient for its function [178]. In the recent study using fluorescence labeling, it has been shown that Y RNAs associate with unreplicated euchromatin in late G1 phase, then disassociate from replicated DNA. Pull-down experiments revealed that they interact with ORC and other pre-RC components. Therefore, a “catch and release” mechanism was proposed for Y RNAs and the Y RNAs accordingly served as another licensing factor for replication initiation [179]. In addition, evidence from *Xenopus* and zebrafish embryos suggests that the midblastula transition (MBT) defines the onset of Y RNA-dependent DNA replication. The Y RNA is not required for DNA replication before MBT, but is necessary for replication and associates with the chromatin in an ORC-dependent fashion after MBT [180].

### G-quadruplex RNA

Epstein-Barr virus (EBV) is an oncogenic herpesvirus that can infect human cells and stay in the host as an episome, which replicates once per cell cycle. EBV utilizes EBV-encoded protein EBNA1 (Epstein–Barr nuclear antigen 1) to recruit host ORC to its origin, *oriP*, in order to replicate its DNA [181-186]. The N terminal domain of EBNA1 has two linking regions 1 and 2 (LR1 and LR2) that interact with ORC, and deletion of LR1 and LR2 from EBNA1 eliminates its ORC binding ability [187]. Sequence analysis of LR1 and LR2 regions shows that they both have arginine- and glycine-rich motifs (RG-rich motifs) [187]. Substitution of arginines or glycines to alanines in LR2 region abolishes ORC association, indicating that the RG-rich motif plays a pivotal role in ORC-EBNA1 association [187]. Interestingly, RNase A but not DNase I disrupts ORC-EBNA1 interaction, suggesting that the recruitment of ORC to EBNA1 is RNA dependent [187]. EBNA1 displays RNA binding ability [188,189], and has a RG-rich motif that is known for its RNA binding activity [190]. Taken together, these indicate an RNA-dependent recruitment of ORC to EBNA1 by the N-terminal LR1 and LR2 regions of EBNA1.

Further analyses reveal that this RNA dependency is from the G-quadruplex RNA [187,191]. A G-quartet is formed by four guanines in a square arrangement via hydrogen bonds, and a G-quadruplex is formed by two or more stacked G-quartets [192]. The involvement of G-quadruplex RNA in ORC-EBNA1 association is demonstrated by introducing a G-quadruplex-interacting compound, BRACO-19, which abolishes EBNA1 recruitment of ORC and inhibits EBNA1-dependent replication of *oriP*[191].

The emergence of non-coding RNAs from these studies implies the existence of additional factors assisting ORC for origin targeting and replication licensing. Intriguingly, upon RNase A treatment, a fraction of ORC is released from chromatin, indicating that the ORC association with chromatin can be partially stabilized by RNA [187]. Therefore, it is highly possible that some structured RNAs mediate ORC recruitment to certain origins. These findings reinforce the important role of non-coding RNAs in the regulation of replication initiation.

## Concluding remarks

**Figure 1:**
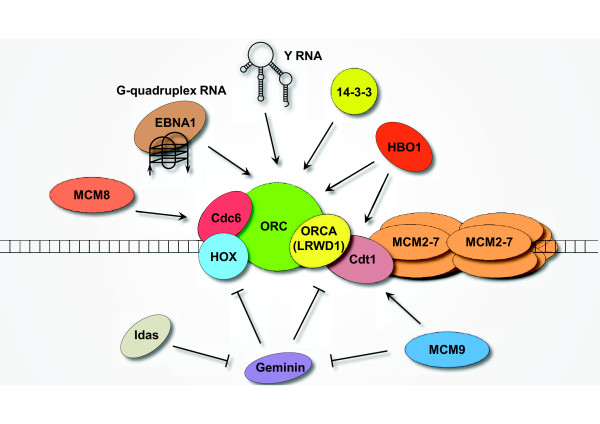
**Emerging players in the assembly of pre-replicative complex (pre-RC). **The classical model of pre-RC assembly involves the ordered loading of ORC, Cdc6, Cdt1 and MCM2-7 onto replication origins. The Cdt1 inhibitor, Geminin associates with Cdt1 outside G1 phase of the cell cycle and prevents the re-assembly of pre-RC. Several ORC interacting proteins (ORCA/LRWD1, 14-3-3) and non-coding RNAs (G-quadruplex RNA, Y RNA) have been found to be involved in regulating ORC binding to origins. MCM8 interacts with Cdc6 and is required for Cdc6 chromatin loading. HBO1 and MCM9 facilitate Cdt1 activity by antagonizing Geminin and modulating Cdt1-Geminin stoichiometry respectively. In addition, HOX and Idas interacts with Geminin and may control Geminin’s dual functions in proliferation-differentiation determination.

**Figure 2:**
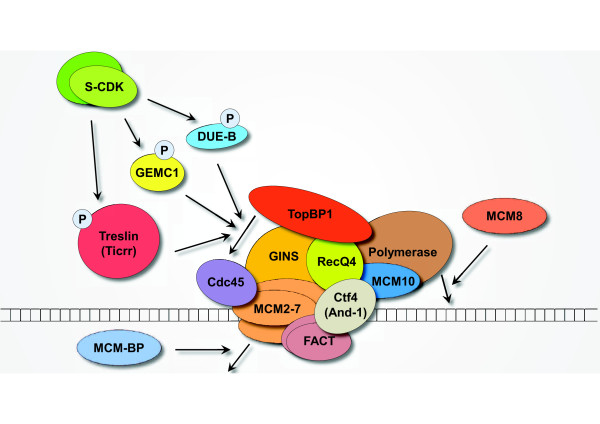
**Emerging players in the assembly of pre-initiation complex (pre-IC) and replisome progression complex (RPC). **Other than classical factors in pre-IC and RPC (Cdc45, MCM2-7, GINS, RecQ4, TopBP1, and DNA polymerase), a number of novel factors have recently been identified. DUE-B, GEMC1, and Treslin/Ticrr all interact with TopBP1 and Cdc45, and phosphorylation of these three proteins (possibly by S phase CDK) are all required for the TopBP1-dependent activation of Cdc45 on chromatin. MCM10, Ctf4/And-1, and FACT connect MCM2-7 helicase activity with DNA polymerase activity and facilitate replisome progression. In addition, MCM-BP regulates replication-coupled MCM2-7 helicase dissociation from chromatin, whereas MCM8 regulates the chromatin assembly of DNA polymerase. (For simple illustration, only one MCM2-7 complex and related factors are presented here).

Recently, several novel members have joined the family of pre-RC (Figure 1) and pre-IC (Figure 2), mostly discovered from proteomic screens/analyses and bioinformatic predictions; at the same time, many classic factors, including HBO1, 14-3-3, HOX, MCM10, FACT, Ctf4, and Y RNAs, were merited with additional roles in replication.

It is notable that different players exhibit diverse conservation levels across the organisms. For some proteins like Ctf4, their functions are faithfully reproduced in all model organisms investigated. For some novel players, such as ORCA/LRWD1, whether orthologs exist or have conserved functions in other eukaryotic species is still an open question. For other factors like MCM9, disparate observations have been made in different systems, which could either reflect technical difference, or indicate real evolutionary divergence.

It is also noteworthy that many of these factors are involved in other non-replication events, evident from ORCA/LRWD1 in heterochromatin silencing [20], 14-3-3 in various signaling pathways [40,41], HOX in developmental control [53-57], FACT in transcription elongation [145,146], Ctf4 in sister chromatid cohesion [159], and Treslin/Ticrr in G2/M checkpoint [130]. It is possible that these factors may have replication-independent functions, or may couple replication to additional processes to form a regulatory network.

What we know is probably only the tip of the iceberg. For instance, the complete *in vitro* reconstitution of DNA replication initiation machinery or sub-complexes using purified proteins has so far been unsuccessful, indicating that there are still important players missing from the orchestra. Investigations in other less-studied species may lead us to some valuable clues. In *Tetrahymena thermophila*, ORC has a 26T RNA component that is involved in ribosomal DNA origin recognition via complementary base pairing [193]. It will be interesting to find out whether a similar functional player exists in other model organisms. On the other hand, with the increasing genome size and complexity of protein network from lower to higher eukaryotes, how timely control and dynamic regulation of replication are achieved also need further elucidation. As reported from a recent study using a novel hybrid CHO cell/*Xenopus* egg extracts, a pre-restriction point inhibitor exists for licensing. Other than ruling out pre-RC components, Geminin, HBO1 or CDK, the nature of this inhibitor was not characterized [194]. Therefore, exploring new model systems and utilizing innovative techniques, along with mechanistic studies on existing factors, will help us probe for the missing links and mechanisms, toward a better grasp of the final truth on how eukaryotes initiate DNA replication.

## Competing interests

The authors declare that they have no competing interests.

## Authors’ contributions

ZS performed literature review and drafted the manuscript. SGP revised and corrected the manuscript. Both authors read and approved the final manuscript.

## References

[B1] Sclafani RA Holzen TM Cell cycle regulation of DNA replication Annu Rev Genet 2007 41 237 280 10.1146/annurev.genet.41.110306.130308 17630848 PMC2292467

[B2] Dutta A Bell SP Initiation of DNA replication in eukaryotic cells Annu Rev Cell Dev Biol 1997 13 293 332 10.1146/annurev.cellbio.13.1.293 9442876

[B3] Kelly TJ Brown GW Regulation of chromosome replication Annu Rev Biochem 2000 69 829 880 10.1146/annurev.biochem.69.1.829 10966477

[B4] Bell SP Dutta A DNA replication in eukaryotic cells Annu Rev Biochem 2002 71 333 374 10.1146/annurev.biochem.71.110601.135425 12045100

[B5] DePamphilis ML The ’ORC cycle’: a novel pathway for regulating eukaryotic DNA replication Gene 2003 310 1 15 12801628 10.1016/s0378-1119(03)00546-8

[B6] Bell SP The origin recognition complex: from simple origins to complex functions Genes Dev 2002 16 659 672 10.1101/gad.969602 11914271

[B7] Newlon CS Yeast chromosome replication and segregation Microbiol Rev 1988 52 568 601 3070325 10.1128/mr.52.4.568-601.1988 PMC373164

[B8] Bell SP Stillman B ATP-dependent recognition of eukaryotic origins of DNA replication by a multiprotein complex Nature 1992 357 128 134 10.1038/357128a0 1579162

[B9] Mendez J Stillman B Chromatin association of human origin recognition complex, cdc6, and minichromosome maintenance proteins during the cell cycle: assembly of prereplication complexes in late mitosis Mol Cell Biol 2000 20 8602 8612 10.1128/MCB.20.22.8602-8612.2000 11046155 PMC102165

[B10] Tye BK MCM proteins in DNA replication Annu Rev Biochem 1999 68 649 686 10.1146/annurev.biochem.68.1.649 10872463

[B11] Labib K How do Cdc7 and cyclin-dependent kinases trigger the initiation of chromosome replication in eukaryotic cells? Genes Dev 2010 24 1208 1219 10.1101/gad.1933010 20551170 PMC2885657

[B12] Nishitani H Lygerou Z Control of DNA replication licensing in a cell cycle Genes to cells: devoted to molecular & cellular mechanisms 2002 7 523 534 10.1046/j.1365-2443.2002.00544.x 12059957

[B13] Feng H Kipreos ET Preventing DNA re-replication–divergent safeguards in yeast and metazoa Cell Cycle 2003 2 431 434 12963835

[B14] DePamphilis ML Cell cycle dependent regulation of the origin recognition complex Cell Cycle 2005 4 70 79 10.4161/cc.4.1.1333 15611627

[B15] McGarry TJ Kirschner MW Geminin, an inhibitor of DNA replication, is degraded during mitosis Cell 1998 93 1043 1053 10.1016/S0092-8674(00)81209-X 9635433

[B16] Melixetian M Helin K Geminin: a major DNA replication safeguard in higher eukaryotes Cell Cycle 2004 3 1002 1004 15280663

[B17] Shen Z Sathyan KM Geng Y Zheng R Chakraborty A Freeman B Wang F Prasanth KV Prasanth SG A WD-repeat protein stabilizes ORC binding to chromatin Mol Cell 2010 40 99 111 10.1016/j.molcel.2010.09.021 20932478 PMC5201136

[B18] Shen Z Chakraborty A Jain A Giri S Ha T Prasanth KV Prasanth SG Dynamic association of ORCA with prereplicative complex components regulates DNA replication initiation Mol Cell Biol 2012 32 3107 3120 10.1128/MCB.00362-12 22645314 PMC3434513

[B19] Bartke T Vermeulen M Xhemalce B Robson SC Mann M Kouzarides T Nucleosome-interacting proteins regulated by DNA and histone methylation Cell 2010 143 470 484 10.1016/j.cell.2010.10.012 21029866 PMC3640253

[B20] Chan KM Zhang Z Leucine-rich Repeat and WD Repeat-containing Protein 1 Is Recruited to Pericentric Heterochromatin by Trimethylated Lysine 9 of Histone H3 and Maintains Heterochromatin Silencing J Biol Chem 2012 287 15024 15033 10.1074/jbc.M111.337980 22427655 PMC3340258

[B21] Neer EJ Schmidt CJ Nambudripad R Smith TF The ancient regulatory-protein family of WD-repeat proteins Nature 1994 371 297 300 10.1038/371297a0 8090199

[B22] Smith TF Gaitatzes C Saxena K Neer EJ The WD repeat: a common architecture for diverse functions Trends Biochem Sci 1999 24 181 185 10.1016/S0968-0004(99)01384-5 10322433

[B23] Li D Roberts R WD-repeat proteins: structure characteristics, biological function, and their involvement in human diseases Cell Mol Life Sci 2001 58 2085 2097 10.1007/PL00000838 11814058 PMC11337334

[B24] Migliori V Mapelli M Guccione E On WD40 proteins: Propelling our knowledge of transcriptional control? Epigenetics 2012 7 8 10.4161/epi.21140 PMC3427277 22810296

[B25] Shen Z Prasanth SG Orc2 protects ORCA from ubiquitin-mediated degradation Cell Cycle 2012 11 19 10.4161/cc.11.1.18633 22935713 PMC3478309

[B26] Vermeulen M Eberl HC Matarese F Marks H Denissov S Butter F Lee KK Olsen JV Hyman AA Stunnenberg HG Mann M Quantitative interaction proteomics and genome-wide profiling of epigenetic histone marks and their readers Cell 2010 142 967 980 10.1016/j.cell.2010.08.020 20850016

[B27] Chakraborty A Shen Z Prasanth SG “ORCanization” on heterochromatin: linking DNA replication initiation to chromatin organization Epigenetics 2011 6 665 670 10.4161/epi.6.6.16179 21586903

[B28] Prasanth SG Prasanth KV Siddiqui K Spector DL Stillman B Human Orc2 localizes to centrosomes, centromeres and heterochromatin during chromosome inheritance EMBO J 2004 23 2651 2663 10.1038/sj.emboj.7600255 15215892 PMC449767

[B29] Ohta S Bukowski-Wills JC Sanchez-Pulido L Alves Fde L Wood L Chen ZA Platani M Fischer L Hudson DF Ponting CP The protein composition of mitotic chromosomes determined using multiclassifier combinatorial proteomics Cell 2010 142 810 821 10.1016/j.cell.2010.07.047 20813266 PMC2982257

[B30] Jaco I Canela A Vera E Blasco MA Centromere mitotic recombination in mammalian cells J Cell Biol 2008 181 885 892 10.1083/jcb.200803042 18541703 PMC2426939

[B31] Reddel RR Alternative lengthening of telomeres, telomerase, and cancer Cancer Lett 2003 194 155 162 10.1016/S0304-3835(02)00702-4 12757973

[B32] Matsuoka S Ballif BA Smogorzewska A McDonald ER 3rd Hurov KE Luo J Bakalarski CE Zhao Z Solimini N Lerenthal Y ATM and ATR substrate analysis reveals extensive protein networks responsive to DNA damage Science 2007 316 1160 1166 10.1126/science.1140321 17525332

[B33] Teng YN Liao MH Lin YB Kuo PL Kuo TY Expression of lrwd1 in mouse testis and its centrosomal localization Int J Androl 2010 33 832 840 10.1111/j.1365-2605.2009.01038.x 20180869

[B34] Iizuka M Stillman B Histone acetyltransferase HBO1 interacts with the ORC1 subunit of the human initiator protein J Biol Chem 1999 274 23027 23034 10.1074/jbc.274.33.23027 10438470

[B35] Iizuka M Matsui T Takisawa H Smith MM Regulation of replication licensing by acetyltransferase Hbo1 Mol Cell Biol 2006 26 1098 1108 10.1128/MCB.26.3.1098-1108.2006 16428461 PMC1347032

[B36] Miotto B Struhl K HBO1 histone acetylase is a coactivator of the replication licensing factor Cdt1 Genes Dev 2008 22 2633 2638 10.1101/gad.1674108 18832067 PMC2559906

[B37] Miotto B Struhl K HBO1 histone acetylase activity is essential for DNA replication licensing and inhibited by Geminin Mol Cell 2010 37 57 66 10.1016/j.molcel.2009.12.012 20129055 PMC2818871

[B38] Wong PG Glozak MA Cao TV Vaziri C Seto E Alexandrow M Chromatin unfolding by Cdt1 regulates MCM loading via opposing functions of HBO1 and HDAC11-geminin Cell Cycle 2010 9 4351 4363 10.4161/cc.9.21.13596 20980834 PMC3055186

[B39] Miotto B Struhl K JNK1 phosphorylation of Cdt1 inhibits recruitment of HBO1 histone acetylase and blocks replication licensing in response to stress Mol Cell 2011 44 62 71 10.1016/j.molcel.2011.06.021 21856198 PMC3190045

[B40] Aitken A 14-3-3 proteins: a historic overview Semin Cancer Biol 2006 16 162 172 10.1016/j.semcancer.2006.03.005 16678438

[B41] Morrison DK The 14-3-3 proteins: integrators of diverse signaling cues that impact cell fate and cancer development Trends Cell Biol 2009 19 16 23 10.1016/j.tcb.2008.10.003 19027299 PMC3073487

[B42] Novac O Alvarez D Pearson CE Price GB Zannis-Hadjopoulos M The human cruciform-binding protein, CBP, is involved in DNA replication and associates in vivo with mammalian replication origins J Biol Chem 2002 277 11174 11183 10.1074/jbc.M107902200 11805087

[B43] Alvarez D Novac O Callejo M Ruiz MT Price GB Zannis-Hadjopoulos M 14-3-3sigma is a cruciform DNA binding protein and associates in vivo with origins of DNA replication J Cell Biochem 2002 87 194 207 12244572 10.1002/jcb.10294

[B44] Zannis-Hadjopoulos M Novac O Alvarez D Price GB 14-3-3s are DNA-replication proteins Biochem Soc Trans 2002 30 397 401 12196102 10.1042/bst0300397

[B45] Callejo M Alvarez D Price GB Zannis-Hadjopoulos M The 14-3-3 protein homologues from Saccharomyces cerevisiae, Bmh1p and Bmh2p, have cruciform DNA-binding activity and associate in vivo with ARS307 J Biol Chem 2002 277 38416 38423 10.1074/jbc.M202050200 12167636

[B46] Yahyaoui W Callejo M Price GB Zannis-Hadjopoulos M Deletion of the cruciform binding domain in CBP/14-3-3 displays reduced origin binding and initiation of DNA replication in budding yeast BMC Mol Biol 2007 8 27 10.1186/1471-2199-8-27 17430600 PMC1865385

[B47] Yahyaoui W Zannis-Hadjopoulos M 14-3-3 proteins function in the initiation and elongation steps of DNA replication in Saccharomyces cerevisiae J Cell Sci 2009 122 4419 4426 10.1242/jcs.044677 19934224

[B48] Beall EL Manak JR Zhou S Bell M Lipsick JS Botchan MR Role for a Drosophila Myb-containing protein complex in site-specific DNA replication Nature 2002 420 833 837 10.1038/nature01228 12490953

[B49] Beall EL Bell M Georlette D Botchan MR Dm-myb mutant lethality in Drosophila is dependent upon mip130: positive and negative regulation of DNA replication Genes Dev 2004 18 1667 1680 10.1101/gad.1206604 15256498 PMC478189

[B50] Calvi BR Byrnes BA Kolpakas AJ Conservation of epigenetic regulation, ORC binding and developmental timing of DNA replication origins in the genus Drosophila Genetics 2007 177 1291 1301 10.1534/genetics.107.070862 18039868 PMC2147948

[B51] Reeves R Nissen MS The A.T-DNA-binding domain of mammalian high mobility group I chromosomal proteins. A novel peptide motif for recognizing DNA structure J Biol Chem 1990 265 8573 8582 1692833

[B52] Thomae AW Pich D Brocher J Spindler MP Berens C Hock R Hammerschmidt W Schepers A Interaction between HMGA1a and the origin recognition complex creates site-specific replication origins Proc Natl Acad Sci U S A 2008 105 1692 1697 10.1073/pnas.0707260105 18234858 PMC2234206

[B53] Gehring WJ Homeo boxes in the study of development Science 1987 236 1245 1252 10.1126/science.2884726 2884726

[B54] Krumlauf R Hox genes in vertebrate development Cell 1994 78 191 201 10.1016/0092-8674(94)90290-9 7913880

[B55] Gehring WJ Qian YQ Billeter M Furukubo-Tokunaga K Schier AF Resendez-Perez D Affolter M Otting G Wuthrich K Homeodomain-DNA recognition Cell 1994 78 211 223 10.1016/0092-8674(94)90292-5 8044836

[B56] Gehring WJ Affolter M Burglin T Homeodomain proteins Annu Rev Biochem 1994 63 487 526 10.1146/annurev.bi.63.070194.002415 7979246

[B57] Favier B Dolle P Developmental functions of mammalian Hox genes Mol Hum Reprod 1997 3 115 131 10.1093/molehr/3.2.115 9239717

[B58] Luo L Yang X Takihara Y Knoetgen H Kessel M The cell-cycle regulator geminin inhibits Hox function through direct and polycomb-mediated interactions Nature 2004 427 749 753 10.1038/nature02305 14973489

[B59] Salsi V Ferrari S Ferraresi R Cossarizza A Grande A Zappavigna V HOXD13 binds DNA replication origins to promote origin licensing and is inhibited by geminin Mol Cell Biol 2009 29 5775 5788 10.1128/MCB.00509-09 19703996 PMC2772751

[B60] Zhou B Liu C Xu Z Zhu G Structural basis for homeodomain recognition by the cell-cycle regulator Geminin Proc Natl Acad Sci U S A 2012 21 21 10.1073/pnas.1200874109 PMC3384217 22615398

[B61] Pefani DE Dimaki M Spella M Karantzelis N Mitsiki E Kyrousi C Symeonidou IE Perrakis A Taraviras S Lygerou Z Idas, a novel phylogenetically conserved geminin-related protein, binds to geminin and is required for cell cycle progression J Biol Chem 2011 286 23234 23246 10.1074/jbc.M110.207688 21543332 PMC3123090

[B62] Shreeram S Sparks A Lane DP Blow JJ Cell type-specific responses of human cells to inhibition of replication licensing Oncogene 2002 21 6624 6632 10.1038/sj.onc.1205910 12242660 PMC3605503

[B63] Tachibana KE Gonzalez MA Guarguaglini G Nigg EA Laskey RA Depletion of licensing inhibitor geminin causes centrosome overduplication and mitotic defects EMBO Rep 2005 6 1052 1057 10.1038/sj.embor.7400527 16179947 PMC1371027

[B64] Seo S Kroll KL Geminin’s double life: chromatin connections that regulate transcription at the transition from proliferation to differentiation Cell Cycle 2006 5 374 379 10.4161/cc.5.4.2438 16479171

[B65] Kroll KL Geminin in embryonic development: coordinating transcription and the cell cycle during differentiation Front Biosci 2007 12 1395 1409 10.2741/2156 17127390

[B66] Luo L Kessel M Geminin coordinates cell cycle and developmental control Cell Cycle 2004 3 711 714 15153800

[B67] Maine GT Sinha P Tye BK Mutants of S. cerevisiae defective in the maintenance of minichromosomes Genetics 1984 106 365 385 6323245 10.1093/genetics/106.3.365 PMC1224244

[B68] Neuwald AF Aravind L Spouge JL Koonin EV AAA+: A class of chaperone-like ATPases associated with the assembly, operation, and disassembly of protein complexes Genome Res 1999 9 27 43 9927482

[B69] Gozuacik D Chami M Lagorce D Faivre J Murakami Y Poch O Biermann E Knippers R Brechot C Paterlini-Brechot P Identification and functional characterization of a new member of the human Mcm protein family: hMcm8 Nucleic Acids Res 2003 31 570 579 10.1093/nar/gkg136 12527764 PMC140502

[B70] Johnson EM Kinoshita Y Daniel DC A new member of the MCM protein family encoded by the human MCM8 gene, located contrapodal to GCD10 at chromosome band 20p12.3-13 Nucleic Acids Res 2003 31 2915 2925 10.1093/nar/gkg395 12771218 PMC156728

[B71] Volkening M Hoffmann I Involvement of human MCM8 in prereplication complex assembly by recruiting hcdc6 to chromatin Mol Cell Biol 2005 25 1560 1568 10.1128/MCB.25.4.1560-1568.2005 15684404 PMC548026

[B72] Kinoshita Y Johnson EM Gordon RE Negri-Bell H Evans MT Coolbaugh J Rosario-Peralta Y Samet J Slusser E Birkenbach MP Daniel DC Colocalization of MCM8 and MCM7 with proteins involved in distinct aspects of DNA replication Microsc Res Tech 2008 71 288 297 10.1002/jemt.20553 18072282

[B73] Maiorano D Cuvier O Danis E Mechali M MCM8 is an MCM2-7-related protein that functions as a DNA helicase during replication elongation and not initiation Cell 2005 120 315 328 10.1016/j.cell.2004.12.010 15707891

[B74] Crevel G Hashimoto R Vass S Sherkow J Yamaguchi M Heck MM Cotterill S Differential requirements for MCM proteins in DNA replication in Drosophila S2 cells PLoS One 2007 2 e833 10.1371/journal.pone.0000833 17786205 PMC1950684

[B75] Oehlmann M Score AJ Blow JJ The role of Cdc6 in ensuring complete genome licensing and S phase checkpoint activation J Cell Biol 2004 165 181 190 10.1083/jcb.200311044 15096526 PMC2172031

[B76] Yoshida K Identification of a novel cell-cycle-induced MCM family protein MCM9 Biochem Biophys Res Commun 2005 331 669 674 10.1016/j.bbrc.2005.03.222 15850810

[B77] Lutzmann M Maiorano D Mechali M Identification of full genes and proteins of MCM9, a novel, vertebrate-specific member of the MCM2-8 protein family Gene 2005 362 51 56 16226853 10.1016/j.gene.2005.07.031

[B78] Lutzmann M Mechali M MCM9 binds Cdt1 and is required for the assembly of prereplication complexes Mol Cell 2008 31 190 200 10.1016/j.molcel.2008.07.001 18657502

[B79] Lutzmann M Mechali M How to load a replicative helicase onto chromatin: a more and more complex matter during evolution Cell Cycle 2009 8 1309 1313 10.4161/cc.8.9.8216 19342892

[B80] Hartford SA Luo Y Southard TL Min IM Lis JT Schimenti JC Minichromosome maintenance helicase paralog MCM9 is dispensible for DNA replication but functions in germ-line stem cells and tumor suppression Proc Natl Acad Sci U S A 2011 108 17702 17707 10.1073/pnas.1113524108 21987787 PMC3203795

[B81] Nishimura K Ishiai M Horikawa K Fukagawa T Takata M Takisawa H Kanemaki MT Mcm8 and Mcm9 Form a Complex that Functions in Homologous Recombination Repair Induced by DNA Interstrand Crosslinks Mol Cell 2012 47 511 522 10.1016/j.molcel.2012.05.047 22771115

[B82] Lutzmann M Grey C Traver S Ganier O Maya-Mendoza A Ranisavljevic N Bernex F Nishiyama A Montel N Gavois E MCM8- and MCM9-Deficient Mice Reveal Gametogenesis Defects and Genome Instability Due to Impaired Homologous Recombination Mol Cell 2012 47 523 534 10.1016/j.molcel.2012.05.048 22771120

[B83] Dumas LB Lussky JP McFarland EJ Shampay J New temperature-sensitive mutants of Saccharomyces cerevisiae affecting DNA replication Mol Gen Genet 1982 187 42 46 10.1007/BF00384381 6761543

[B84] Solomon NA Wright MB Chang S Buckley AM Dumas LB Gaber RF Genetic and molecular analysis of DNA43 and DNA52: two new cell-cycle genes in Saccharomyces cerevisiae Yeast 1992 8 273 289 10.1002/yea.320080405 1514326

[B85] Merchant AM Kawasaki Y Chen Y Lei M Tye BK A lesion in the DNA replication initiation factor Mcm10 induces pausing of elongation forks through chromosomal replication origins in Saccharomyces cerevisiae Mol Cell Biol 1997 17 3261 3271 9154825 10.1128/mcb.17.6.3261 PMC232179

[B86] Liu Y Richards TA Aves SJ Ancient diversification of eukaryotic MCM DNA replication proteins BMC Evol Biol 2009 9 60 10.1186/1471-2148-9-60 19292915 PMC2667178

[B87] Fien K Cho YS Lee JK Raychaudhuri S Tappin I Hurwitz J Primer utilization by DNA polymerase alpha-primase is influenced by its interaction with Mcm10p J Biol Chem 2004 279 16144 16153 10.1074/jbc.M400142200 14766746

[B88] Okorokov AL Waugh A Hodgkinson J Murthy A Hong HK Leo E Sherman MB Stoeber K Orlova EV Williams GH Hexameric ring structure of human MCM10 DNA replication factor EMBO Rep 2007 8 925 930 10.1038/sj.embor.7401064 17823614 PMC2002553

[B89] Robertson PD Warren EM Zhang H Friedman DB Lary JW Cole JL Tutter AV Walter JC Fanning E Eichman BF Domain architecture and biochemical characterization of vertebrate Mcm10 J Biol Chem 2008 283 3338 3348 18065420 10.1074/jbc.M706267200 PMC2753450

[B90] Warren EM Vaithiyalingam S Haworth J Greer B Bielinsky AK Chazin WJ Eichman BF Structural basis for DNA binding by replication initiator Mcm10 Structure 2008 16 1892 1901 10.1016/j.str.2008.10.005 19081065 PMC2636851

[B91] Warren EM Huang H Fanning E Chazin WJ Eichman BF Physical interactions between Mcm10, DNA, and DNA polymerase alpha J Biol Chem 2009 284 24662 24672 10.1074/jbc.M109.020438 19608746 PMC2782055

[B92] Eisenberg S Korza G Carson J Liachko I Tye BK Novel DNA binding properties of the Mcm10 protein from Saccharomyces cerevisiae J Biol Chem 2009 284 25412 25420 10.1074/jbc.M109.033175 19605346 PMC2757242

[B93] Cook CR Kung G Peterson FC Volkman BF Lei M A novel zinc finger is required for Mcm10 homocomplex assembly J Biol Chem 2003 278 36051 36058 10.1074/jbc.M306049200 12844493

[B94] Homesley L Lei M Kawasaki Y Sawyer S Christensen T Tye BK Mcm10 and the MCM2-7 complex interact to initiate DNA synthesis and to release replication factors from origins Genes Dev 2000 14 913 926 10783164 PMC316538

[B95] Gambus A Jones RC Sanchez-Diaz A Kanemaki M van Deursen F Edmondson RD Labib K GINS maintains association of Cdc45 with MCM in replisome progression complexes at eukaryotic DNA replication forks Nat Cell Biol 2006 8 358 366 10.1038/ncb1382 16531994

[B96] Liang DT Forsburg SL Characterization of Schizosaccharomyces pombe mcm7(+) and cdc23(+) (MCM10) and interactions with replication checkpoints Genetics 2001 159 471 486 11606526 10.1093/genetics/159.2.471 PMC1461838

[B97] Hart EA Bryant JA Moore K Aves SJ Fission yeast Cdc23 interactions with DNA replication initiation proteins Curr Genet 2002 41 342 348 10.1007/s00294-002-0316-9 12185500

[B98] Lee JK Seo YS Hurwitz J The Cdc23 (Mcm10) protein is required for the phosphorylation of minichromosome maintenance complex by the Dfp1-Hsk1 kinase Proc Natl Acad Sci U S A 2003 100 2334 2339 10.1073/pnas.0237384100 12604790 PMC151341

[B99] Zhu W Ukomadu C Jha S Senga T Dhar SK Wohlschlegel JA Nutt LK Kornbluth S Dutta A Mcm10 and And-1/CTF4 recruit DNA polymerase alpha to chromatin for initiation of DNA replication Genes Dev 2007 21 2288 2299 10.1101/gad.1585607 17761813 PMC1973143

[B100] Apger J Reubens M Henderson L Gouge CA Ilic N Zhou HH Christensen TW Multiple functions for Drosophila Mcm10 suggested through analysis of two Mcm10 mutant alleles Genetics 2010 185 1151 1165 10.1534/genetics.110.117234 20498296 PMC2927746

[B101] Izumi M Yanagi K Mizuno T Yokoi M Kawasaki Y Moon KY Hurwitz J Yatagai F Hanaoka F The human homolog of Saccharomyces cerevisiae Mcm10 interacts with replication factors and dissociates from nuclease-resistant nuclear structures in G(2) phase Nucleic Acids Res 2000 28 4769 4777 10.1093/nar/28.23.4769 11095689 PMC115166

[B102] van Deursen F Sengupta S De Piccoli G Sanchez-Diaz A Labib K Mcm10 associates with the loaded DNA helicase at replication origins and defines a novel step in its activation EMBO J 2012 31 2195 2206 2110.1038/emboj.2012.2169 22433841 10.1038/emboj.2012.69 PMC3343467

[B103] Ricke RM Bielinsky AK Mcm10 regulates the stability and chromatin association of DNA polymerase-alpha Mol Cell 2004 16 173 185 10.1016/j.molcel.2004.09.017 15494305

[B104] Ricke RM Bielinsky AK A conserved Hsp10-like domain in Mcm10 is required to stabilize the catalytic subunit of DNA polymerase-alpha in budding yeast J Biol Chem 2006 281 18414 18425 10.1074/jbc.M513551200 16675460

[B105] Chattopadhyay S Bielinsky AK Human Mcm10 regulates the catalytic subunit of DNA polymerase-alpha and prevents DNA damage during replication Mol Biol Cell 2007 18 4085 4095 10.1091/mbc.E06-12-1148 17699597 PMC1995709

[B106] Lee C Liachko I Bouten R Kelman Z Tye BK Alternative mechanisms for coordinating polymerase alpha and MCM helicase Mol Cell Biol 2010 30 423 435 10.1128/MCB.01240-09 19917723 PMC2798462

[B107] Wang J Wu R Lu Y Liang C Ctf4p facilitates Mcm10p to promote DNA replication in budding yeast Biochem Biophys Res Commun 2010 395 336 341 10.1016/j.bbrc.2010.04.006 20381454

[B108] Taylor M Moore K Murray J Aves SJ Price C Mcm10 interacts with Rad4/Cut5(TopBP1) and its association with origins of DNA replication is dependent on Rad4/Cut5(TopBP1) DNA Repair (Amst) 2011 10 1154 1163 10.1016/j.dnarep.2011.09.001 21945095

[B109] Wohlschlegel JA Dhar SK Prokhorova TA Dutta A Walter JC Xenopus Mcm10 binds to origins of DNA replication after Mcm2-7 and stimulates origin binding of Cdc45 Mol Cell 2002 9 233 240 10.1016/S1097-2765(02)00456-2 11864598

[B110] Gregan J Lindner K Brimage L Franklin R Namdar M Hart EA Aves SJ Kearsey SE Fission yeast Cdc23/Mcm10 functions after pre-replicative complex formation to promote Cdc45 chromatin binding Mol Biol Cell 2003 14 3876 3887 10.1091/mbc.E03-02-0090 12972571 PMC196582

[B111] Sawyer SL Cheng IH Chai W Tye BK Mcm10 and Cdc45 cooperate in origin activation in Saccharomyces cerevisiae J Mol Biol 2004 340 195 202 10.1016/j.jmb.2004.04.066 15201046

[B112] Heller RC Kang S Lam WM Chen S Chan CS Bell SP Eukaryotic origin-dependent DNA replication in vitro reveals sequential action of DDK and S-CDK kinases Cell 2011 146 80 91 10.1016/j.cell.2011.06.012 21729781 PMC3204357

[B113] Watase G Takisawa H Kanemaki MT Mcm10 plays a role in functioning of the eukaryotic replicative DNA helicase, Cdc45-Mcm-GINS Curr Biol 2012 22 343 349 10.1016/j.cub.2012.01.023 22285032

[B114] Kanke M Kodama Y Takahashi TS Nakagawa T Masukata H Mcm10 plays an essential role in origin DNA unwinding after loading of the CMG components EMBO J 2012 31 2182 2194 2110.1038/emboj.2012.2168 22433840 10.1038/emboj.2012.68 PMC3343466

[B115] Sakwe AM Nguyen T Athanasopoulos V Shire K Frappier L Identification and characterization of a novel component of the human minichromosome maintenance complex Mol Cell Biol 2007 27 3044 3055 10.1128/MCB.02384-06 17296731 PMC1899943

[B116] Nishiyama A Frappier L Mechali M MCM-BP regulates unloading of the MCM2-7 helicase in late S phase Genes Dev 2011 25 165 175 10.1101/gad.614411 21196493 PMC3022262

[B117] Ding L Forsburg SL Schizosaccharomyces pombe minichromosome maintenance-binding protein (MCM-BP) antagonizes MCM helicase J Biol Chem 2011 286 32918 32930 10.1074/jbc.M111.282541 21813639 PMC3190919

[B118] Li JJ Schnick J Hayles J MacNeill SA Purification and functional inactivation of the fission yeast MCM(MCM-BP) complex FEBS Lett 2011 585 3850 3855 10.1016/j.febslet.2011.10.033 22036784

[B119] Takahashi N Lammens T Boudolf V Maes S Yoshizumi T De Jaeger G Witters E Inze D De Veylder L The DNA replication checkpoint aids survival of plants deficient in the novel replisome factor ETG1 EMBO J 2008 27 1840 1851 10.1038/emboj.2008.107 18528439 PMC2486427

[B120] Jagannathan M Sakwe AM Nguyen T Frappier L The MCM-associated protein MCM-BP is important for human nuclear morphology J Cell Sci 2012 125 133 143 10.1242/jcs.089938 22250201

[B121] Nguyen T Jagannathan M Shire K Frappier L Interactions of the human MCM-BP protein with MCM complex components and Dbf4 PLoS One 2012 7 e35931 10.1371/journal.pone.0035931 22540012 PMC3335088

[B122] Takahashi N Quimbaya M Schubert V Lammens T Vandepoele K Schubert I Matsui M Inze D Berx G De Veylder L The MCM-binding protein ETG1 aids sister chromatid cohesion required for postreplicative homologous recombination repair PLoS Genet 2010 6 e1000817 10.1371/journal.pgen.1000817 20090939 PMC2806904

[B123] Casper JM Kemp MG Ghosh M Randall GM Vaillant A Leffak M The c-myc DNA-unwinding element-binding protein modulates the assembly of DNA replication complexes in vitro J Biol Chem 2005 280 13071 13083 15653697 10.1074/jbc.M404754200

[B124] Kemp M Bae B Yu JP Ghosh M Leffak M Nair SK Structure and function of the c-myc DNA-unwinding element-binding protein DUE-B J Biol Chem 2007 282 10441 10448 10.1074/jbc.M609632200 17264083

[B125] Chowdhury A Liu G Kemp M Chen X Katrangi N Myers S Ghosh M Yao J Gao Y Bubulya P Leffak M The DNA unwinding element binding protein DUE-B interacts with Cdc45 in preinitiation complex formation Mol Cell Biol 2010 30 1495 1507 10.1128/MCB.00710-09 20065034 PMC2832489

[B126] Balestrini A Cosentino C Errico A Garner E Costanzo V GEMC1 is a TopBP1-interacting protein required for chromosomal DNA replication Nat Cell Biol 2010 12 484 491 10.1038/ncb2050 20383140 PMC2875115

[B127] Piergiovanni G Costanzo V GEMC1 is a novel TopBP1-interacting protein involved in chromosomal DNA replication Cell Cycle 2010 9 3662 3666 10.4161/cc.9.18.13060 20855966 PMC3047794

[B128] Kumagai A Shevchenko A Dunphy WG Treslin collaborates with TopBP1 in triggering the initiation of DNA replication Cell 2010 140 349 359 10.1016/j.cell.2009.12.049 20116089 PMC2857569

[B129] Kumagai A Shevchenko A Dunphy WG Direct regulation of Treslin by cyclin-dependent kinase is essential for the onset of DNA replication J Cell Biol 2011 193 995 1007 10.1083/jcb.201102003 21646402 PMC3115804

[B130] Sansam CL Cruz NM Danielian PS Amsterdam A Lau ML Hopkins N Lees JA A vertebrate gene, ticrr, is an essential checkpoint and replication regulator Genes Dev 2010 24 183 194 10.1101/gad.1860310 20080954 PMC2807353

[B131] Sangrithi MN Bernal JA Madine M Philpott A Lee J Dunphy WG Venkitaraman AR Initiation of DNA replication requires the RECQL4 protein mutated in Rothmund-Thomson syndrome Cell 2005 121 887 898 10.1016/j.cell.2005.05.015 15960976

[B132] Matsuno K Kumano M Kubota Y Hashimoto Y Takisawa H The N-terminal noncatalytic region of Xenopus RecQ4 is required for chromatin binding of DNA polymerase alpha in the initiation of DNA replication Mol Cell Biol 2006 26 4843 4852 10.1128/MCB.02267-05 16782873 PMC1489170

[B133] Van Hatten RA Tutter AV Holway AH Khederian AM Walter JC Michael WM The Xenopus Xmus101 protein is required for the recruitment of Cdc45 to origins of DNA replication J Cell Biol 2002 159 541 547 10.1083/jcb.200207090 12438414 PMC2173091

[B134] Hashimoto Y Tsujimura T Sugino A Takisawa H The phosphorylated C-terminal domain of Xenopus Cut5 directly mediates ATR-dependent activation of Chk1 Genes Cells 2006 11 993 1007 10.1111/j.1365-2443.2006.00998.x 16923121

[B135] Yan S Lindsay HD Michael WM Direct requirement for Xmus101 in ATR-mediated phosphorylation of Claspin bound Chk1 during checkpoint signaling J Cell Biol 2006 173 181 186 10.1083/jcb.200601076 16618813 PMC2063809

[B136] Fu YV Walter JC DNA replication: metazoan Sld3 steps forward Curr Biol 2010 20 R515 R517 10.1016/j.cub.2010.05.033 20620904

[B137] Sanchez-Pulido L Diffley JF Ponting CP Homology explains the functional similarities of Treslin/Ticrr and Sld3 Curr Biol 2010 20 R509 R510 10.1016/j.cub.2010.05.021 20620901

[B138] Mueller AC Keaton MA Dutta A DNA replication: mammalian Treslin-TopBP1 interaction mirrors yeast Sld3-Dpb11 Curr Biol 2011 21 R638 R640 10.1016/j.cub.2011.07.004 21855008 PMC3523092

[B139] Boos D Sanchez-Pulido L Rappas M Pearl LH Oliver AW Ponting CP Diffley JF Regulation of DNA replication through Sld3-Dpb11 interaction is conserved from yeast to humans Curr Biol 2011 21 1152 1157 10.1016/j.cub.2011.05.057 21700459

[B140] Wang Z Kim E Leffak M Xu YJ Treslin, DUE-B, and GEMC1 cannot complement Sld3 mutants in fission yeast FEMS Yeast Res 2012 12 486 490 410.1111/j.1567-1364.2012.00794.x 22380713 10.1111/j.1567-1364.2012.00794.x PMC3336028

[B141] Thangavel S Mendoza-Maldonado R Tissino E Sidorova JM Yin J Wang W Monnat RJ Jr Falaschi A Vindigni A Human RECQ1 and RECQ4 helicases play distinct roles in DNA replication initiation Mol Cell Biol 2010 30 1382 1396 10.1128/MCB.01290-09 20065033 PMC2832491

[B142] Xu X Liu Y Dual DNA unwinding activities of the Rothmund-Thomson syndrome protein, RECQ4 EMBO J 2009 28 568 577 10.1038/emboj.2009.13 19177149 PMC2657580

[B143] Xu X Rochette PJ Feyissa EA Su TV Liu Y MCM10 mediates RECQ4 association with MCM2-7 helicase complex during DNA replication EMBO J 2009 28 3005 3014 10.1038/emboj.2009.235 19696745 PMC2760112

[B144] Im JS Ki SH Farina A Jung DS Hurwitz J Lee JK Assembly of the Cdc45-Mcm2-7-GINS complex in human cells requires the Ctf4/And-1, RecQL4, and Mcm10 proteins Proc Natl Acad Sci U S A 2009 106 15628 15632 10.1073/pnas.0908039106 19805216 PMC2747170

[B145] Orphanides G LeRoy G Chang CH Luse DS Reinberg D FACT, a factor that facilitates transcript elongation through nucleosomes Cell 1998 92 105 116 10.1016/S0092-8674(00)80903-4 9489704

[B146] LeRoy G Orphanides G Lane WS Reinberg D Requirement of RSF and FACT for transcription of chromatin templates in vitro Science 1998 282 1900 1904 9836642 10.1126/science.282.5395.1900

[B147] Orphanides G Wu WH Lane WS Hampsey M Reinberg D The chromatin-specific transcription elongation factor FACT comprises human SPT16 and SSRP1 proteins Nature 1999 400 284 288 10.1038/22350 10421373

[B148] Formosa T Eriksson P Wittmeyer J Ginn J Yu Y Stillman DJ Spt16-Pob3 and the HMG protein Nhp6 combine to form the nucleosome-binding factor SPN EMBO J 2001 20 3506 3517 10.1093/emboj/20.13.3506 11432837 PMC125512

[B149] Brewster NK Johnston GC Singer RA A bipartite yeast SSRP1 analog comprised of Pob3 and Nhp6 proteins modulates transcription Mol Cell Biol 2001 21 3491 3502 10.1128/MCB.21.10.3491-3502.2001 11313475 PMC100271

[B150] Okuhara K Ohta K Seo H Shioda M Yamada T Tanaka Y Dohmae N Seyama Y Shibata T Murofushi H A DNA unwinding factor involved in DNA replication in cell-free extracts of Xenopus eggs Curr Biol 1999 9 341 350 10.1016/S0960-9822(99)80160-2 10209116

[B151] Wittmeyer J Formosa T The Saccharomyces cerevisiae DNA polymerase alpha catalytic subunit interacts with Cdc68/Spt16 and with Pob3, a protein similar to an HMG1-like protein Mol Cell Biol 1997 17 4178 4190 9199353 10.1128/mcb.17.7.4178 PMC232271

[B152] Wittmeyer J Joss L Formosa T Spt16 and Pob3 of Saccharomyces cerevisiae form an essential, abundant heterodimer that is nuclear, chromatin-associated, and copurifies with DNA polymerase alpha Biochemistry 1999 38 8961 8971 10.1021/bi982851d 10413469

[B153] VanDemark AP Blanksma M Ferris E Heroux A Hill CP Formosa T The structure of the yFACT Pob3-M domain, its interaction with the DNA replication factor RPA, and a potential role in nucleosome deposition Mol Cell 2006 22 363 374 10.1016/j.molcel.2006.03.025 16678108

[B154] Tan BC Chien CT Hirose S Lee SC Functional cooperation between FACT and MCM helicase facilitates initiation of chromatin DNA replication EMBO J 2006 25 3975 3985 10.1038/sj.emboj.7601271 16902406 PMC1560368

[B155] Tan BC Liu H Lin CL Lee SC Functional cooperation between FACT and MCM is coordinated with cell cycle and differential complex formation J Biomed Sci 2010 17 11 10.1186/1423-0127-17-11 20156367 PMC2848000

[B156] Abe T Sugimura K Hosono Y Takami Y Akita M Yoshimura A Tada S Nakayama T Murofushi H Okumura K The histone chaperone facilitates chromatin transcription (FACT) protein maintains normal replication fork rates J Biol Chem 2011 286 30504 30512 10.1074/jbc.M111.264721 21757688 PMC3162410

[B157] Kundu LR Seki M Watanabe N Murofushi H Furukohri A Waga S Score AJ Blow JJ Horikoshi M Enomoto T Tada S Biphasic chromatin binding of histone chaperone FACT during eukaryotic chromatin DNA replication Biochim Biophys Acta 2011 1813 1129 1136 10.1016/j.bbamcr.2011.01.002 21232560 PMC3428913

[B158] Kouprina N Kroll E Bannikov V Bliskovsky V Gizatullin R Kirillov A Shestopalov B Zakharyev V Hieter P Spencer F CTF4 (CHL15) mutants exhibit defective DNA metabolism in the yeast Saccharomyces cerevisiae Mol Cell Biol 1992 12 5736 5747 1341195 10.1128/mcb.12.12.5736-5747.1992 PMC360513

[B159] Hanna JS Kroll ES Lundblad V Spencer FA Saccharomyces cerevisiae CTF18 and CTF4 are required for sister chromatid cohesion Mol Cell Biol 2001 21 3144 3158 10.1128/MCB.21.9.3144-3158.2001 11287619 PMC86942

[B160] Suter B Tong A Chang M Yu L Brown GW Boone C Rine J The origin recognition complex links replication, sister chromatid cohesion and transcriptional silencing in Saccharomyces cerevisiae Genetics 2004 167 579 591 10.1534/genetics.103.024851 15238513 PMC1470908

[B161] Gambus A van Deursen F Polychronopoulos D Foltman M Jones RC Edmondson RD Calzada A Labib K A key role for Ctf4 in coupling the MCM2-7 helicase to DNA polymerase alpha within the eukaryotic replisome EMBO J 2009 28 2992 3004 10.1038/emboj.2009.226 19661920 PMC2760104

[B162] Tanaka H Katou Y Yagura M Saitoh K Itoh T Araki H Bando M Shirahige K Ctf4 coordinates the progression of helicase and DNA polymerase alpha Genes Cells 2009 14 807 820 10.1111/j.1365-2443.2009.01310.x 19496828

[B163] Gosnell JA Christensen TW Drosophila Ctf4 is essential for efficient DNA replication and normal cell cycle progression BMC Mol Biol 2011 12 13 10.1186/1471-2199-12-13 21470422 PMC3082215

[B164] Bermudez VP Farina A Tappin I Hurwitz J Influence of the human cohesion establishment factor Ctf4/AND-1 on DNA replication J Biol Chem 2010 285 9493 9505 10.1074/jbc.M109.093609 20089864 PMC2843200

[B165] Hodgson B Calzada A Labib K Mrc1 and Tof1 regulate DNA replication forks in different ways during normal S phase Mol Biol Cell 2007 18 3894 3902 10.1091/mbc.E07-05-0500 17652453 PMC1995724

[B166] Hayano M Kanoh Y Matsumoto S Masai H Mrc1 marks early-firing origins and coordinates timing and efficiency of initiation in fission yeast Mol Cell Biol 2011 31 2380 2391 10.1128/MCB.01239-10 21518960 PMC3133423

[B167] Wawrousek KE Fortini BK Polaczek P Chen L Liu Q Dunphy WG Campbell JL Xenopus DNA2 is a helicase/nuclease that is found in complexes with replication proteins And-1/Ctf4 and Mcm10 and DSB response proteins Nbs1 and ATM Cell Cycle 2010 9 1156 1166 10.4161/cc.9.6.11049 20237432 PMC3059328

[B168] Guler GD Fanning E The replisome: a nanomachine or a dynamic dance of protein partners? Cell Cycle 2010 9 1680 1681 20448477

[B169] Hendrick JP Wolin SL Rinke J Lerner MR Steitz JA Ro small cytoplasmic ribonucleoproteins are a subclass of La ribonucleoproteins: further characterization of the Ro and La small ribonucleoproteins from uninfected mammalian cells Mol Cell Biol 1981 1 1138 1149 6180298 10.1128/mcb.1.12.1138-1149.1981 PMC369740

[B170] Pruijn GJ Slobbe RL Van Venrooij WJ Structure and function of La and Ro RNPs Mol Biol Rep 1990 14 43 48 10.1007/BF00360410 2194109

[B171] Pruijn GJ Wingens PA Peters SL Thijssen JP van Venrooij WJ Ro RNP associated Y RNAs are highly conserved among mammals Biochim Biophys Acta 1993 1216 395 401 10.1016/0167-4781(93)90006-Y 7505620

[B172] Farris AD O’Brien CA Harley JB Y3 is the most conserved small RNA component of Ro ribonucleoprotein complexes in vertebrate species Gene 1995 154 193 198 10.1016/0378-1119(94)00823-B 7534247

[B173] Mosig A Guofeng M Stadler BM Stadler PF Evolution of the vertebrate Y RNA cluster Theory Biosci 2007 126 9 14 10.1007/s12064-007-0003-y 18087752

[B174] Perreault J Perreault JP Boire G Ro-associated Y RNAs in metazoans: evolution and diversification Mol Biol Evol 2007 24 1678 1689 10.1093/molbev/msm084 17470436

[B175] Christov CP Gardiner TJ Szuts D Krude T Functional requirement of noncoding Y RNAs for human chromosomal DNA replication Mol Cell Biol 2006 26 6993 7004 10.1128/MCB.01060-06 16943439 PMC1592862

[B176] Langley AR Chambers H Christov CP Krude T Ribonucleoprotein particles containing non-coding Y RNAs, Ro60, La and nucleolin are not required for Y RNA function in DNA replication PLoS One 2010 5 e13673 10.1371/journal.pone.0013673 21060685 PMC2965120

[B177] Krude T Christov CP Hyrien O Marheineke K Y RNA functions at the initiation step of mammalian chromosomal DNA replication J Cell Sci 2009 122 2836 2845 10.1242/jcs.047563 19657016

[B178] Gardiner TJ Christov CP Langley AR Krude T A conserved motif of vertebrate Y RNAs essential for chromosomal DNA replication RNA 2009 15 1375 1385 10.1261/rna.1472009 19474146 PMC2704080

[B179] Zhang AT Langley AR Christov CP Kheir E Shafee T Gardiner TJ Krude T Dynamic interaction of Y RNAs with chromatin and initiation proteins during human DNA replication J Cell Sci 2011 124 2058 2069 10.1242/jcs.086561 21610089 PMC3104036

[B180] Collart C Christov CP Smith JC Krude T The midblastula transition defines the onset of Y RNA-dependent DNA replication in Xenopus laevis Mol Cell Biol 2011 31 3857 3870 10.1128/MCB.05411-11 21791613 PMC3165727

[B181] Adams A Replication of latent Epstein-Barr virus genomes in Raji cells J Virol 1987 61 1743 1746 3033303 10.1128/jvi.61.5.1743-1746.1987 PMC254169

[B182] Yates JL Guan N Epstein-Barr virus-derived plasmids replicate only once per cell cycle and are not amplified after entry into cells J Virol 1991 65 483 488 1845903 10.1128/jvi.65.1.483-488.1991 PMC240543

[B183] Chaudhuri B Xu H Todorov I Dutta A Yates JL Human DNA replication initiation factors, ORC and MCM, associate with oriP of Epstein-Barr virus Proc Natl Acad Sci U S A 2001 98 10085 10089 10.1073/pnas.181347998 11517328 PMC56919

[B184] Dhar SK Yoshida K Machida Y Khaira P Chaudhuri B Wohlschlegel JA Leffak M Yates J Dutta A Replication from oriP of Epstein-Barr virus requires human ORC and is inhibited by geminin Cell 2001 106 287 296 10.1016/S0092-8674(01)00458-5 11509178

[B185] Schepers A Ritzi M Bousset K Kremmer E Yates JL Harwood J Diffley JF Hammerschmidt W Human origin recognition complex binds to the region of the latent origin of DNA replication of Epstein-Barr virus EMBO J 2001 20 4588 4602 10.1093/emboj/20.16.4588 11500385 PMC125560

[B186] Lindner SE Sugden B The plasmid replicon of Epstein-Barr virus: mechanistic insights into efficient, licensed, extrachromosomal replication in human cells Plasmid 2007 58 1 12 10.1016/j.plasmid.2007.01.003 17350094 PMC2562867

[B187] Norseen J Thomae A Sridharan V Aiyar A Schepers A Lieberman PM RNA-dependent recruitment of the origin recognition complex EMBO J 2008 27 3024 3035 10.1038/emboj.2008.221 18946490 PMC2585170

[B188] Snudden DK Hearing J Smith PR Grasser FA Griffin BE EBNA-1, the major nuclear antigen of Epstein-Barr virus, resembles ‘RGG’ RNA binding proteins EMBO J 1994 13 4840 4847 7957053 10.1002/j.1460-2075.1994.tb06810.x PMC395423

[B189] Lu CC Wu CW Chang SC Chen TY Hu CR Yeh MY Chen JY Chen MR Epstein-Barr virus nuclear antigen 1 is a DNA-binding protein with strong RNA-binding activity J Gen Virol 2004 85 2755 2765 10.1099/vir.0.80239-0 15448336

[B190] Burd CG Dreyfuss G Conserved structures and diversity of functions of RNA-binding proteins Science 1994 265 615 621 10.1126/science.8036511 8036511

[B191] Norseen J Johnson FB Lieberman PM Role for G-quadruplex RNA binding by Epstein-Barr virus nuclear antigen 1 in DNA replication and metaphase chromosome attachment J Virol 2009 83 10336 10346 10.1128/JVI.00747-09 19656898 PMC2753104

[B192] Huppert JL Four-stranded nucleic acids: structure, function and targeting of G-quadruplexes Chem Soc Rev 2008 37 1375 1384 10.1039/b702491f 18568163

[B193] Mohammad MM Donti TR Sebastian Yakisich J Smith AG Kapler GM Tetrahymena ORC contains a ribosomal RNA fragment that participates in rDNA origin recognition EMBO J 2007 26 5048 5060 10.1038/sj.emboj.7601919 18007594 PMC2140106

[B194] Sasaki T Li A Gillespie PJ Blow JJ Gilbert DM Evidence for a mammalian late-G1 phase inhibitor of replication licensing distinct from geminin or Cdk activity Nucleus 2011 2 455 464 10.4161/nucl.2.5.17859 21983086 PMC3322585

